# Hand before foot? Cortical somatotopy suggests manual dexterity is primitive and evolved independently of bipedalism

**DOI:** 10.1098/rstb.2012.0417

**Published:** 2013-11-19

**Authors:** Teruo Hashimoto, Kenichi Ueno, Akitoshi Ogawa, Takeshi Asamizuya, Chisato Suzuki, Kang Cheng, Michio Tanaka, Miki Taoka, Yoshiaki Iwamura, Gen Suwa, Atsushi Iriki

**Affiliations:** 1 Laboratory for Symbolic Cognitive Development, RIKEN Brain Science Institute, 2-1 Hirosawa, Wako, Saitama 351-0198, Japan; 2 Support Unit for Functional Magnetic Resonance Imaging, RIKEN Brain Science Institute, 2-1 Hirosawa, Wako, Saitama 351-0198, Japan; 3 Department of Physiology, Toho University School of Medicine, 5-21-16 Omorinishi, Ota-ku, Tokyo 143-8540, Japan; 4 The University Museum, The University of Tokyo, 3-1 Hongo, Bunkyo-ku, Tokyo 113-0033, Japan

**Keywords:** somatotopic representation, electrophysiology, neuroimaging, fingers, toes

## Abstract

People have long speculated whether the evolution of bipedalism in early hominins triggered tool use (by freeing their hands) or whether the necessity of making and using tools encouraged the shift to upright gait. Either way, it is commonly thought that one led to the other. In this study, we sought to shed new light on the origins of manual dexterity and bipedalism by mapping the neural representations in the brain of the fingers and toes of living people and monkeys. Contrary to the ‘hand-in-glove’ notion outlined above, our results suggest that adaptations underlying tool use evolved independently of those required for human bipedality. In both humans and monkeys, we found that each finger was represented separately in the primary sensorimotor cortex just as they are physically separated in the hand. This reflects the ability to use each digit independently, as required for the complex manipulation involved in tool use. The neural mapping of the subjects’ toes differed, however. In the monkeys, the somatotopic representation of the toes was fused, showing that the digits function predominantly as a unit in general grasping. Humans, by contrast, had an independent neurological representation of the big toe (hallux), suggesting association with bipedal locomotion. These observations suggest that the brain circuits for the hand had advanced beyond simple grasping, whereas our primate ancestors were still general arboreal quadrupeds. This early adaptation laid the foundation for the evolution of manual dexterity, which was preserved and enhanced in hominins. In hominins, a separate adaptation, involving the neural separation of the big toe, apparently occurred with bipedality. This accords with the known fossil evidence, including the recently reported hominin fossils which have been dated to 4.4 million years ago.

## 1. Introduction

With the advent of genome-wide molecular studies [1,2], chimpanzees have been shown to be genetically the most similar, and phylogenetically closest, to humans (and equally so is the bonobo). The early human ancestral lineage(s) has, therefore, commonly been evaluated with respect to the extant chimpanzee. We have limited information regarding our early ancestors, however, and the evolutionary path of the human hand is not well known.

Recent studies that model the hand of the last common ancestor (LCA) of humans and chimpanzees suggest that it was similar to those of a chimpanzee or a modern African ape [3–5]. If this is correct, it means that some drastic morphological changes occurred in hominins (all species on the human side of the human–chimpanzee split), a view which derives from observations of modern humans and apes, and hominin fossils dated approximately 4.2 million years ago (Ma) and younger. Relative to a chimpanzee-like LCA, species of *Australopithecus* would have had a greatly shortened palm and fingers relative to the thumb, but nevertheless retained aspects of an ape-like hand for arboreal climbing. They lacked features related to advanced tool use, such as a robust thumb and other anatomical changes associated with human-like manual dexterity, widely considered to have occurred after approximately 2.5 Ma [4–7].

**Figure 1.**
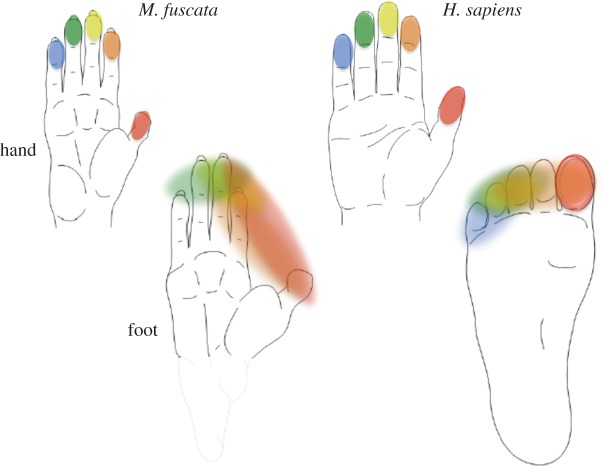
The shape of the hand and foot in two primate species. Both monkey (*Macaca fuscata*, left) and human (*Homo sapiens*, right) have five physically independent fingers (top) and five toes (bottom), although the human foot is irregular in shape. Monkey hand, foot and human hand are similar in shape except for the monkey heel (grey). In addition, human toe I is larger than the lesser toes, whereas monkey toes are similar in size. The fingers were represented independently (colour coded) in the primate somatosensory cortex (SI): I, red; II, orange; III, yellow; IV, green and V, blue. By contrast, the representations of the toes were fused, with the exception of the big toe in humans.

Old World monkeys, however, have hands that are in many respects more similar to the human hand than are those of the chimpanzees, even though they diverged from humans much earlier. In contrast to the hand, the feet of both apes and monkeys differ from the bipedal human foot (figure 1). This paradox of the chimpanzee (and other extant apes) hand being apparently too specialized for the LCA is known in comparative anatomy as ‘the riddle of man's ancestry’ [8]. One possible way to throw new light on this conundrum is to infer limb adaptation and concomitant neurophysiological changes from a more primitive ancestral model than living apes, and to compare the cortical representation of the hands and feet of humans with that of Old World monkeys.

This approach is helped by the recent discovery of a partial skeleton and other well-preserved hand and foot elements [9–11] of approximately 4.4 Ma-old *Ardipithecus* (*Ar.*) *ramidus.* These fossils suggest that hominin limbs evolved directly from those of ancestors with morphologies more similar to quadrupedal Miocene apes and Old World monkeys than to the suspensory-adapted limbs of extant apes [9,10,12]. For example, while the *Ar. ramidus* foot and pelvis exhibit mosaic structures suitable for both climbing and upright bipedal walking [11,13], its hand retains functional features suited for palmigrade quadrupedal postures and arboreal clambering [10,12]. The morphology of the *Ar. ramidus* hand is, in many ways, broadly similar to that of quadrupedal monkeys. This suggests that hominins may not have evolved from a chimpanzee-like ancestor adapted for suspensory locomotion. The suspensory and knuckle-walking hands of extant chimpanzees and gorillas may have emerged independently.

The hands of comparatively well-known hominin ancestors such as *Australopithecus* and early *Homo* have been reported to exhibit individual details of bone features that are similar to those of the African apes [3,4]. However, the actual fossil evidence is highly fragmentary, and the *Ar. ramidus* fossils also share many of these features [10]. Given the extensive non-suspensory trunk and limb morphologies revealed by *Ar. ramidus* [9,10,12], the ape-like morphologies of *Australopithecus* and early *Homo* are probably primitive retentions from a Miocene ape ancestor, rather than *Pan* (chimpanzee)-like. Thus, the human ability to use and make tools may not have required as extensive a modification of the hands as previously thought. From a generalized (non-suspensory) arboreal hand, the development of manual dexterity necessary for hominin tool use would have involved relatively small structural changes. Nor does tool use necessarily lead to body changes related to committed bipedalism; evidence shows that spontaneous stone-tool use occurs frequently in quadrupedal primates such as chimpanzees and capuchin monkeys [14,15].

All this contradicts the notion that manual dexterity evolved as a result of bipedality, which frees the hands from locomotion. Rather, the primate (including human) hand and foot must have evolved at least in part independently [5,16].

**Figure 2.**
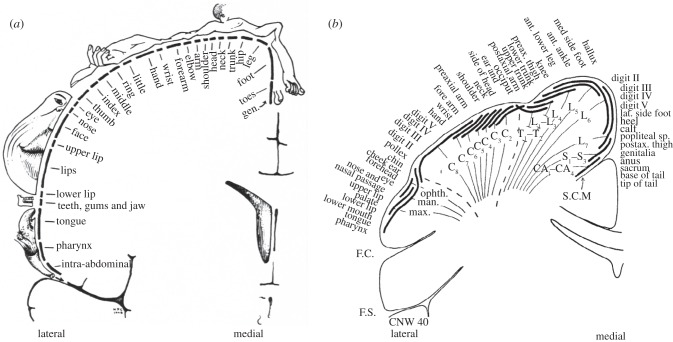
Somatotopy maps of humans (*a*) (adopted from fig. 17 of [19]) and monkeys (*b*) (Adapted from fig. 14 of [18].) Coronal sections of the primary somatosensory cortex (SI) of each primate are shown. A correspondence between the position of the body part and the focal brain area has been reported. The arrangements of body parts in the SI of both species are similar. Five fingers and five toes are depicted.

Comparative neuroscience can provide valuable clues regarding these issues. The neural representation of the body in the primary sensorimotor cortex is somatotopic, mirroring both shape and function. If limb use alters so does the sensory input from the limb to the brain and this, in turn, causes changes in the appropriate part of the cortical ‘map’. The species-specific cortical organization of each mammal is associated with its ecological niche and lifestyle [17]. Increased dexterity enlarges the cortical areas devoted to discrete divisions of limb structures, and textbooks generally describe the cortical representations of the hand and foot in monkeys [18] and humans [19] (figure 2) as having a separate area for each digit. This description may need to be revised in the light of the evidence supporting the differential evolution of limb appendages.

In order to shed light on this issue, we compared finger and toe representations in the primary somatosensory cortex (area 3b, SI) of humans and monkeys (Japanese macaque). We used the most precise neurophysiological procedure available for each species. That is, high-spatial-resolution functional magnetic resonance imaging (fMRI) for humans and electrophysiological recordings from single neurons for monkeys. Although using an identical method for both species would be better for direct comparison, these different methods were used, because single neuron recordings are not possible in humans and monkeys do not accept passive tactile stimulation for fMRI measurement without anaesthesia (which alters brain responses [20,21]), whereas activity of monkey primary visual cortex as shown by electrophyiological recordings is strongly correlated with fMRI signals [22].

We examined three hypotheses.

(a) If finger and toe representations are similar in the two species, then they derived from a common ancestral condition. (b) If they are different, they evolved either independently or sequentially.
The hands and feet of monkeys and the hand of humans have similar general shapes. (a) If the cortical maps of these limbs are also similar, but distinct from that of the human foot, then the neurology reflects general shape and/or their common grasping function. (b) If the cortical maps are dissimilar they reflect more distinct functional characteristics.
(a) If the cortical map of the human hand is different from that of the monkeys’, the human hand evolved significantly to allow uniquely complex use of tools. (b) If the cortical arrangement is similar, then the dexterity of the human hand is an adaptive reuse of an existing mechanism with only minor modifications.

## 2. Methods

### (a) Human fMRI experiments

#### (i) Participants

Four healthy right-handed and right-footed (identified by the Waterloo footedness questionnaire revised) [23] individuals (mean age, 33 years) participated in the fMRI experiment. Two participants (S1 and S4) were male, and two (S2 and S3) were female. The experiment was approved in advance by the Third Research Ethics Committee of RIKEN and was conducted in accordance with the Declaration of Helsinki. Written informed consents were obtained from all subjects prior to the experiment.

#### (ii) Stimulations

The left hand and both feet were studied in each participant. The stimulation device, which was electronically controlled by a computer, was equipped with five independently controllable motors, each of which controlled a carbon rod that was placed in front of the patient table of the scanner (see the electronic supplementary material, figure S1). The subject lay on the patient table in the supine position with his or her feet extending outside the scanner. Vibrotactile stimuli were delivered to each toe through one of the five carbon rods (diameter, 2 mm). Each of the carbon rods was composed of three 96 cm-long subsections and two plastic adjustable 2 cm joints, resulting in a total length of 290±2 cm. For the finger stimulations, the carbon rod was made of four subsections and three plastic joints, with a total length of 387±3 cm. The stimulation end of the carbon rod was covered with a plastic cap. The whole stimulation system was functional inside the magnet room, and we confirmed, during the preliminary scans, that the motors did not result in MRI signal loss or image distortions. The position of the device and the lengths of the five carbon rods were adjusted for each individual, so that the stimulation tip of each carbon rod was approximately 2 mm away from the tip of the volar surface of the digit (toe or finger) to be stimulated. During stimulation, the carbon rod vibrated 4 mm in an anteroposterior direction and pressed perpendicularly onto each digit over approximately 2 mm. In order to exclude kinaesthetic/motor components, the foot or hand that was being stimulated was fixed to a custom-made acrylic board that was fixed to the patient table. The position of the hand or foot could be adjusted and fixed through another board that was attached to the acrylic board, such that the first joints of the digits were fixed and unable to move. The board that was used for fixing the hand was placed beside the subject's waist, and the hand was almost parallel to the body. The foot was fixed in a supine position, so that the toes were perpendicular to the body. Adjacent toes were separated with a sponge pad in order to prevent vibration conduction. The opposing foot was not fixed during stimulation.

The innocuous vibration frequency of 40 Hz was chosen, because Meissner's corpuscle, which has the highest density of mechanical receptors on glabrous skin and which has small receptive fields (RFs), is most sensitive to a 25–40 Hz flutter frequency [24]. Although the data that were collected in this study may have included effects from other mechanoreceptors, mechanical stimulation is generally preferred to electrical stimulation because it represents a natural stimulus.

During the acquisition of the fMRI data, a 10.7 s rest period was followed by a 5 s vibration period. A 5 s stimulation to the skin's surface has been shown to evoke focal and strong neural responses in the primary somatosensory cortex [25]. Each digit was given 12 stimulations, and the stimulation sequence was arranged as a haptic working-memory task. Three fMRI runs were conducted in 2 days: day 1 included the stimulation of the left hand and day 2 included the stimulation of the right and left toes. On day 2, two subjects (S1 and S2) received stimulations to the left toes first, and the other two (S3 and S4) received right-toe stimulations first. The sequences of the stimulations across the three experimental runs were counterbalanced in order to prevent order effects.

#### (iii) Procedures

In order to identify the regions that corresponded to digit movement and digit sensation, localizer scans of voluntary finger and toe movement were conducted prior to the finger experiment. The localizer scan for the fingers was used to confirm the locus of the central sulcus (CS). The scan consisted of alternating blocks of 15 s of movement and 15 s of rest. A vibrotactile stimulation of the left thumb for 1 s was the cue to start the movement, and a stimulation of the left little finger for 1 s was the cue to stop the movement. Two localizer runs were conducted: one for the left fingers (five movement blocks and five rest blocks) and the other for the toes of both feet (five right-toe movement blocks, five left-toe movement blocks and 10 rest blocks). During the localization scans, the toes were not fixed, and the fingers were roughly fixed in such a way that the digits could move.

During the experiment in which individual digits were stimulated, we instructed the subject to perform a working-memory task in order to keep the subject's attention on the tactile stimuli. Enhanced neural activity in the somatosensory cortex resulting from attention to and retention of tactile stimuli has been reported in human neuroimaging and monkey single-neuron studies [25]. In this study, the subject was required to judge whether the stimulation had been delivered to the same digit as the one immediately preceding it by pressing one of two buttons with the right fingers (i.e. button 1 for same and button 2 for different). The subject was instructed not to verbalize the name of the stimulated digit. Therefore, the participants maintained their focus on the tactile sensation in the stimulated digit during the 10.7 s interstimulus period. No visual stimuli were presented, and the subject was instructed to close his or her eyes. Each digit was stimulated 12 times (in total, 60 stimulations on each foot and on the left hand), and successive stimulation (i.e. same) of each digit occurred four times during each experimental run. Stimulations of adjacent digits (I–II, II–I, II–III, III–II, III–IV, IV–III, IV–V and V–IV) occurred three or four times in each experiment, thus amounting to about half of all trials. Each experiment lasted 942 s, plus the 15 s that was allotted to the pre-experimental scan period.

#### (iv) fMRI data acquisition and analysis

Images were collected with a 4-T Varian unity Inova MRI system (Varian NMR Instruments, Palo Alto, CA). A high-resolution three-dimensional fast low-angle shot (FLASH) T1-weighted structural image (inversion time (TI), 0.5 s; repetition time (TR), 110 ms; echo time (TE), 6.2 ms; flip angle (FA), 11°; 256×256×256; voxel size, 1×1×1) was obtained before the functional experiment.

For the localizer scans, a four-segmented T2*-weighted Echo planar imaging (EPI; TR, 1.3 s; TE, 25 ms; FA, 51°) pulse sequence was used, and 13 slices (slice thickness, 3 mm; field of view (FOV), 19.2 × 19.2 cm^2^; matrix size, 64×64; in-plane resolution, 3×3 mm^2^) parallel to the anterior commissure–posterior commissure plane were prescribed in order to cover the toe and finger regions. A total of 140 volumes was acquired for finger localization. In order to localize the right and left toes, 260 volumes were acquired.

Five oblique slices of the EPI images (TR, 2.24 s; TE, 25 ms; FA, 30°; slice thickness, 2.5 mm; FOV, 16 × 16 cm^2^; matrix size, 128 × 128; in-plane resolution, 1.25 × 1.25 mm^2^) were acquired in the three vibrotactile experiments. The first and most anterior slice covered the CS of each hemisphere of the individual subject based on the localizer scan and the high-resolution anatomical image. In this study, we defined area 3b as the region that was located in the posterior bank of the CS, and the slices were aligned to area 3b.

In each vibrotactile experimental run, 428 volumes were collected. A set of low-resolution three-dimensional FLASH T1-weighted images (TI, 0.5 s; TR, 110 ms; TE, 6.2 ms; FA, 11°; matrix size, 128×128×180; voxel size, 1.71×1.71×1.71 mm) with the same scan coordinates as those of the EPI were acquired immediately after the EPI scan. The EPI and T1-weighted images were co-registered and then registered to the high-resolution structural images. Rigid head motion was restricted with a bite bar and sponge pads. Respiration and cardiac signals were recorded simultaneously and used in post-processing to remove physiological fluctuations [26] from the functional images.

The images were pre-processed and analysed with brain voyager QX 2.1 software (Brain Innovation BV, Maastricht, The Netherlands). Functional images were stripped of any linear trends and temporally smoothed with a high-pass filter (1/120 Hz, about 0.0083 Hz). In addition, a two-dimensional motion correction was applied. No spatial normalization or smoothing was applied. The blood oxygen level-dependent signals were modelled with a boxcar function that was convolved with a synthetic haemodynamic response function. The first seven volumes were discarded.

For the localization scans, the neural activation corresponding to right-toe movement was compared with that corresponding to left-toe movement. For the vibrotactile experiments, the neural activations that were recorded during the stimulation of each digit were compared with those recorded during the interstimulus period. Direct comparisons between digits revealed an absence of significant differences. Data that included both correct and incorrect responses in the working-memory task were analysed in order to examine the individual correspondence between the behavioural responses and brain activation. No comparisons between the hemispheres were performed because of the regional differences in scanning coverage. Activations that surpassed the cluster-level significance at a threshold of *p* < 0.001 (uncorrected) and that had a minimum of two voxels were reported. Because of interindividual differences [19], only individual-level analyses were performed.

Three indices were used for the analyses: the distances between the centre-of-gravity coordinates of the activation elicited by the stimulation of each digit, the rates of the overlapped voxels between the activations during the stimulation of adjacent digits and the rates of the non-overlapped areas (NOAs) between adjacent digits. The centre-of-gravity coordinates were calculated with *t*-values, and the coordinates [*x, y, z*] for digit I (thumb per big toe) were defined as the position of the origin [0, 0, 0]. The Euclidean distances between the digits were calculated within each participant with the centre-of-gravity coordinates. The rates of overlapped voxels between adjacent digits, which were defined as the number of overlapped voxels divided by the sum of the voxels between adjacent digits, were calculated.

The mean rates of NOAs of the Gaussian distributions between adjacent digit pairs were calculated. The activation centre was defined as the centre of gravity of the *t*-values of activated voxels for each finger or toe. A three-dimensional Gaussian function was fit to the voxel *t*-values in order to estimate the sharpness of the spatial tuning of the activation in the brain. After the centre of the Gaussian, function was spatially fixed to the centre-of-gravity activation for each finger or toe, the peak amplitude and its full width at half maximum was estimated from the best-fit function. The voxels in the brain tissue and within a 6 mm radius from the centre-of-gravity activation were taken into account. The differences in the tuning curves for adjacent fingers or toes were evaluated based on the one-dimensional profile along the activation peaks of the three-dimensional Gaussian functions. The Gaussian functions for the toes were calculated with both the right- and left-toe pairs. The discriminability index (DI) was defined as the percentage of the NOA between the two one-dimensional Gaussian curves, such that DI = [area 1 + area 2 − 2 × overlap(1,2)]/[area 1 + area 2 − overlap(1,2)], 0 < DI < 1.

### (b) Monkey electrophysiological experiments

Single-unit recordings to explore finger and toe representation of SI were made for three hemispheres (one for the finger representation and two for the toe representation) of two male Japanese monkeys (*Macaca fuscata*) weighing 7.0 and 7.5 kg. Independent representation of each finger has been confirmed previously with penetrations perpendicular to the skull surface and multiple-digit representations have been reported to be scarce in area 3b [27,28]. In this study, individual finger representations were directly reconfirmed and multiple-digit representations were examined with penetrations oblique to the skull surface to cover intra-penetrations areas with perpendicular penetrations. This study was approved by the Animal Research Committee at the Toho University School of Medicine. All animal care and experimental procedures were in accordance with NIH guide for the care and use of laboratory animals.

#### (i) Surgeries for electrophysiological recordings

Surgeries before the single-unit recording were done in two steps. First, four metal bolts to fix the monkey's head to the chair were implanted in the skull under anaesthesia with pentobarbital sodium (30 mg kg^−1^). After the recovery of this surgery, an operation for setting recording chamber (**ϕ** = 2 cm) was done for each monkey. For the animal used in the experiments of the finger region, a trephine opening of about 2 cm diameter was made in the left hemisphere to cover the finger region. A chamber was placed approximately 60° oblique to the skull surface along the direction of CS to allow electrode penetrations to pass through more than one digit representations. The oblique penetrations were applied to record from neurons covering multiple digits and to examine transitions between representations of digits. In case of the toe region, a trephine opening was made over the midline region of the skull and the chamber was placed perpendicularly to the skull surface to explore the toe representing regions of both hemispheres simultaneously. The chambers were fixed to the skull using metal bolts and resins.

#### (ii) Single-unit recordings and identification of the receptive field

Single-unit recordings were done according to the methods described in previous studies [27,28]. Briefly, electrode penetrations were made with a microdrive (Narishige MO-95) using a glass-insulated platinum-iridium microelectrode (impedance, 4–6 M*Ω*). At the ends of selected penetrations, one to three electrolytic lesions were made for later identification and reconstruction of these penetrations histologically.

Neural signals recorded through the electrode were amplified and monitored on an oscilloscope and a sound monitor system. After encountering a single neural activity, identification of the RF was done with somatosensory stimulation to its fingers and toes in both sides of the body. The extents of RFs were examined by various somatosensory stimuli such as light touch, rubbing, tapping and kneading using the experimenter's hands and hand-held instruments.

#### (iii) Histological examination

After the single-unit recording experiments were completed, the monkey was sacrificed with an overdose of pentobarbital and perfused transcardially with 0.9% saline, which was followed by 10% formalin. Before removing the brain from the skull, three guide wires were inserted into each explored region through the microdrive that was attached to the cylinder in order to indicate the orientation of the penetrations. Brain blocks from the finger and toe regions were processed for dehydration and celloidin embedding, and sectioned at 40 µm thickness. The sections from the finger region parallel to the CS and perpendicular to the skull surface were cut through the postcentral gyrus to observe the traces of penetrations. The sections from the toe region were at a right angle to the CS. Every section was stained with cresyl violet, and gliosis around the electrode tracks and electrolytic lesions were looked for carefully for every section. All the trajectories of electrodes were reconstructed and assigned to one of the penetrations with the guidance of its surface location and patterned lesions. The ratio of shrinkage of the brain through the histological procedures was estimated by overall shrinkage of the brain block and the decrease of depth of the lesion site. The depth of recording site of each unit was estimated based on the distance reading of the electrode manipulator during the experiments and the ratio of shrinkage. The borders of the cytoarchitectonic subdivisions were determined in each section with previously published criteria [29] for the toe region, and those with response properties for the finger region [27].

## 3. Results

### (a) Human behaviours

Only the stimulations that were applied to toe I were perfectly isolable and discriminable. The average numbers of correct responses (mean±s.d.) for the five toes (I–V) of the right foot were 1.00±0.00, 0.85±0.07, 0.81±0.07, 0.83±0.11 and 0.94±0.07, respectively, and, for the left foot, these were 1.00±0.00, 0.81±0.10, 0.81±0.07, 0.75±0.13 and 0.88±0.10, respectively. These values included errors that were made when the stimulations were delivered to the same toe in succession. No errors were observed for the big toe, which was in contrast to the less frequent but consistent errors across participants that were observed for the lesser four toes. In lesser toes, toe V was relatively discriminable. In our control task, participants were able to easily identify whether each stimulation had been delivered to the same digit for each of the five fingers of the left hand. The mean accuracies in distinguishing the stimulations that were applied to each digit (the thumb, index, middle, ring and little finger, respectively) were 1.00, 0.96, 1.00, 0.90 and 0.96 (for data for individual subjects, see the electronic supplementary material, table S1).

In order to investigate the potential differences in the accuracy in identifying successive stimulations between the fingers and toes, we used a 3 (limbs: left fingers, right toes and left toes) × 5 (digits) two-way ANOVA with repeated measures. This revealed marginally significant differences between fingers and toes (*F*_2,6_ = 4.58, *p* = 0.06) and significant differences between digits (*F*_4,12_ = 13.27, *p* < 0.0005). A post-hoc analysis (Ryan's method) showed significant differences in accuracy between I–II (*t* = 4.27, *p* < 0.005), I–III (*t* = 4.90, *p* < 0.0001), I–IV (*t* = 4.07, *p* < 0.0005) and V–III (*t* = 2.91, *p* < 0.01) for the right toes and between I–II (*t* = 4.61, *p* < 0.0001), I–III (*t* = 4.90, *p* < 0.0001), I–IV (*t* = 5.43, *p* < 0.0001) and I–V (*t* = 3.44, *p* < 0.005) for the left toes. For the fingers, there were significant differences in accuracy between III–IV (*t* = 3.10, *p* < 0.005) and I–IV (*t* = 3.10, *p* < 0.005). Cumulatively, these results showed that, for both feet, only the great toe was clearly discriminable from the other toes as vibrotactile stimulations were not easily distinguishable between the adjacent lesser toes. In some cases, participants were also unable to discern when the same toe was stimulated serially.

### (b) fMRI results

**Figure 3.**
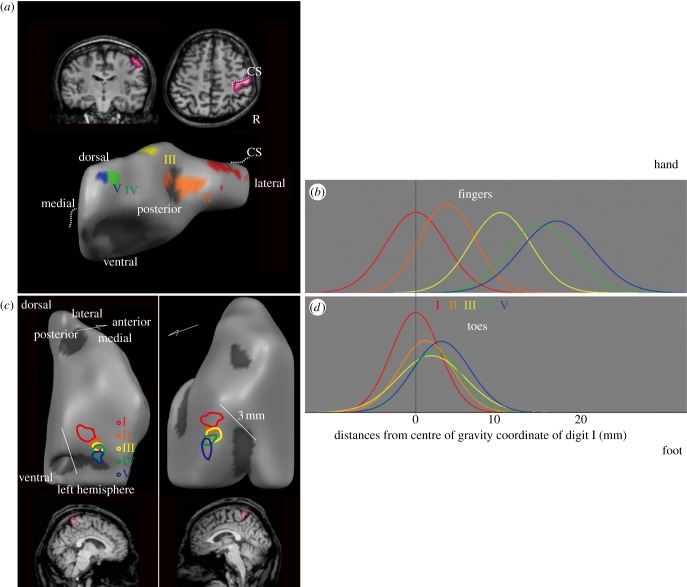
Finger and toe somatotopy in the human primary somatosensory cortex (SI). (*a*) For the hand, transverse and coronal slices of the right hemisphere and the three-dimensional structure of the postcentral gyrus of a single participant (S1) are shown. (*c*) For the foot, the brain regions corresponding to the stimulation of each toe of a single participant (S4, *p* < 0.0006, uncorrected for display purposes) are shown. The region outlined in pink corresponds to the three-dimensional structure. Separate fingers, a big toe and overlapped lesser toes are observed. The digits were colour coded as follows: I, red; II, orange; III, yellow; IV, green and V, blue. The dotted line indicates the central sulcus (CS). R, right. (*b*,*d*) The mean Gaussian distributions for each finger (*n* = 4, (*b*)) and toe (*n* = 8, (*d*)). All fingers and the big toe were mutually separated with a definite statistical threshold, whereas the lesser toes were inseparable.

As has previously been reported [30], we found an orderly shift in the representations for finger I (the thumb) to finger V (the little finger) laterally to medially, anteriorly to posteriorly and ventrally to dorsally (figure 3*a*). In addition, we observed a similar tendency for the toe representations (figure 3*c*; the centre-of-gravity coordinates of the activation that was elicited by stimulation to each toe or finger are given in the electronic supplementary material, table S2).

A 3 (limbs: left fingers, right toes and left toes) × 4 (adjacent digit pairs: digits I and II (I–II), digits II–III (II–III), digits III and IV (III–IV) and digits IV–V (IV–V)) two-way ANOVA showed that the toes of both feet overlapped each other more than did the left fingers (*F*_2,6_ = 6.03, *p* < 0.05; electronic supplementary material, table S3). No significant differences were found between adjacent pairs, but we did observe an interaction between the limbs and the adjacent pairs (*F*_6,18_ = 6.11, *p* < 0.005). The number of overlapped voxels between digits II and III was significantly greater for the left (*t* = 4.86, *p* < 0.00001) and right (*t* = 2.58, *p* < 0.05) toes than for the left fingers. In addition, we observed a larger number of overlapped voxels between digits III and IV for the left (*t* = 3.77, *p* < 0.001) and right (*t* = 3.17, *p* < 0.005) toes than for the left fingers.

Significant differences were observed in the Euclidean distances between the centre-of-gravity coordinates of the adjacent digits of the left fingers, right toes and left toes (*F*_2,6_ = 35.66, *p* < 0.0005; electronic supplementary material, figure S2 and table S4); however, no differences were found between the digits. There were significantly shorter distances between the right toes (*t* = 7.09, *p* < 0.0005) and left toes (*t* = 7.51, *p* < 0.0005) than between the left fingers.

The NOAs of adjacent fingers (figure 3*b*) were significantly larger than those for adjacent toes (figure 3*d*; *F*_1,10_ = 27.43, *p* < 0.0005). The NOA between toes I and II was as large as that between fingers I and II (see the electronic supplementary material, table S5). Importantly, the NOA between toes I and II was significantly larger than that between the remaining adjacent pairs of toes (I–II versus II–III: *t* = 3.35, *p* < 0. 005; I–II versus III–IV: *t* = 2.82, *p* < 0.01; I–II versus IV–V: *t* = 2.67, *p* < 0.05). These results indicated that the cortical representations of the lesser toes overlapped, whereas the representation of the big toe was distinct.

### (c) Monkey electrophysiology

**Figure 4.**
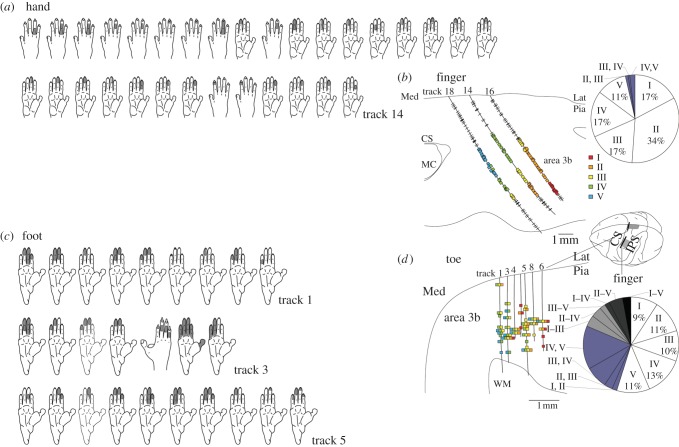
Receptive field properties of single neurons in monkey primary somatosensory cortex (SI) area 3b. The receptive fields are shown of the neurons recorded along an electrode penetration (track) for the fingers and toes. The extent of the skin's receptive fields is shaded in grey (*a,c*). Separate representations and receptive fields of the fingers were observed in the neurons. By contrast, fused representations of the toes were detected in half of the neurons. The reconstructed neuron recording sites are indicated in (*b,d*). Toes I–V were ordered, roughly, laterally to medially. The rectangles along the penetrations display the neurons with receptive fields on the digit. The paralleled rectangles show a neuron with receptive fields on multiple digits. The circular graph in the panel on the right shows the distribution of the receptive fields of the neurons of fingers/toes; the coloured area depicts multiple-digit neurons. Two left hemispheres of two monkeys were studied and those were rendered on a cortical surface for display purpose. CS, central sulcus; Lat, lateral; MC, motor cortex; Med, medial; Pia, pia mater; IPS, intra-parietal sulcus; WM, white matter.

Only data from area 3b were analysed. No neuronal responses were observed to ipsilateral stimulations on the fingers and toes in all isolated units recorded in three hemispheres. Figure 4 shows the reconstructed electrode trajectories and RF locations of isolated neurons in the finger and toe regions (of the left hemispheres of two animals).

As shown in figure 4*b*, three electrodes were successfully penetrated through the representations from digit I to V. Among the isolated neurons along those three penetrations, RFs were determined for a total of 164 neurons. Each recording electrode was penetrated diagonally to the surface, so that more than one digit representation was recorded along one penetration. For example, along track 14 in figure 4*a,b*, 15 neurons responding to tactile stimulation on digit IV were observed continuously after area one neurons were recorded though the RF locations varied within the digit. Subsequently, six neurons with RFs on digit III were observed consecutively (except for one digit IV neuron), and then digit II neurons were recorded. As a result, RFs moved from digit IV to II in a mediolateral progression of recording sites. Similar shift of RF locations along the penetrations was observed in two other penetrations (tracks 16 and 18 in figure 4*b*).

The circle graph in figure 4*b* shows percentage of each digit representation based on 164 identified neurons recorded in the finger region. As shown in this figure, RFs of almost all neurons (158 out of 164) were confined to single-digit, and the remaining six had RFs covered double-digits (paralleled rectangles in figure 4*b*); two neurons of digits II and III, one neuron of digits III and IV, and 3 neurons of digits IV and V. The majority of single-digit representation as well as the mediolateral progression of RF locations shown in figure 4*a,b* clearly demonstrated the existence of somatotopic organization of finger representation in area 3b as was already reported by Iwamura *et al* [27,28] using conscious macaque monkeys.

On the contrary to the finger region, only rough somatotopic organizations were observed in the toe region as shown in figure 4*c*,*d*. Neurons with RFs on digit V, IV and III were identified for the medial penetrations (track 1 and 3), whereas those of digit III, II, and I were identified for the lateral penetrations (track 6). The reason for this less clear somatotopic arrangement in the toe region is that only half of the neurons had RFs in the single digit (59 out of 108; 54%) as depicted in the circle graph in figure 4*d.* The single-digit neurons were associated with the five different toes in similar proportions. Among 49 multiple-digit neurons, 29 were double-digit, 10 were triple-digit, seven were quad-digit, and three were all-digit. Similar results were found for the monkey's left toes (right hemisphere, data not shown).

## 4. Discussion

Our results indicate that the SI somatotopic representation for each finger is separate in both monkeys and humans. The representation of all five toes is fused in monkeys, whereas, in humans, the representation of the four smaller toes is fused but the big toe is separate. These findings suggest that there should be a major revision of the classic somatotopic map of digits that depicts equally separated fingers and toes in humans and monkeys [18,19].

Further, our results show that functional characteristics of the digits are reflected in the brain to a greater extent than previously believed. The similarities between humans and monkeys seen in the somatotopic separation of the fingers suggest a neural/functional pattern that originated before their evolutionary bifurcation, whereas the independent representation of the big toe in humans indicates a neural/functional adaptation that occurred after their genetic paths diverged. This suggests that the human hand and foot followed distinct and independent evolutionary trajectories. These findings are consistent with the increasing hominin fossil evidence that we further discuss below.

### (a) Pinching fingers and grasping toes of monkeys

The separate SI somatotopic representations of fingers in monkeys [27] suggest their dexterous use of individual fingers. Fine somatosensory resolution is critical to hand dexterity, because the sensory feedback from fingers contributes to adaptive precision grip [31]. Monkeys can use tools as an extension of their arms [32], whereas chimpanzees lack tool-related grips, such as forceful gripping, precision handling and power squeezing, owing to their long fingers and short thumbs [7,33,34]. Rather, the chimpanzee hand is elongated and hook-like, and effective in hanging from trees. In chimpanzees (and bonobos and gorillas), the backs of the middle fingers contact the ground (or large branches) during quadrupedal knuckle-walking. By contrast, monkeys dorsiflex their hands and place their palms and/or extended fingers on the ground or branch during quadrupedal walking. As in humans, monkeys can use the thumb both in precision grips, such as pinching an object between the tip of the thumb and other fingers, and in power grips [35]. Although similarities in somatotopic organization between monkeys [18] and chimpanzees [36] have been reported, this remains to be conclusively examined. It is possible that differences in their hand use are associated with different cortical organizations.

Both apes and monkeys have a grasping foot with an opposable hallux (big toe). The common ancestral primate is thought to have had a grasping foot with an opposable hallux [37] for the power grip required in body support and propulsion. Its hand and fingers, by contrast, were specialized for precise control in exploring the surrounding space and in ‘steering’ [38]. The results of this study appear to reflect these functional differences, indicating that they had already been established in the common ancestor of humans and macaques. This is despite the latter's *quadrumanous* morphology, in which all four limbs had similar general distal configurations with flat-nails (not claws) and opposable thumb or big toe. Monkeys combine a foot derived for arboreal activities and a hand capable of tool use.

### (b) Functional adaptation of the human hand and foot

After the bifurcation of the Old World monkey and human (and extant ape) lineages, the essential design of the hand did not change much in either lineage or taxonomic group. Indeed, existing knowledge suggests that the graphical plan of SI finger representation is identical, whereas the area occupied by the finger representations relative to the whole SI, while lacking quantitative accuracy, appears similar, between monkeys and humans (figure 2). By contrast, the human big toe function became independent of the lateral toes, whereas monkeys retained the all-fused somatotopic condition associated with whole-foot power grasping in arboreal propulsion.

The grasping foot that lacks the independent use of toes is not as sensitive or dexterous as the hand and, consistent with the fused-toe somatotopic representations that were observed in this study, monkeys have difficulty distinguishing tactile stimulations between one toe and another [39]. However, in monkeys trained to walk upright, pressure beneath their big toe during bipedal locomotion was observed to increase [40]. Recent studies have suggested the importance of sensory feedback from the sole, including the big toe, in the control of balance during bipedality [41,42], and this is supported by the experiments done with the human volunteers in this study on touch discrimination in toes and fingers and working-memory. The subjects made no errors when asked to detect and remember a touch to their big toe, but they made consistent (though infrequent) errors when asked to do the same for the other toes. By contrast, the participants easily identified whether each stimulus had been delivered to the same fingertips. Thus, the human big toe, but not the lateral toes, exhibit functional independence equivalent to that observed in the individual fingers. Alternatively, the somatotopic space of the four lateral toes may have been degenerated or sacrificed in order to maximize the space for the big toe.

These results suggest a function-dependent separation of finger and toe somatotopic representations. We hypothesize that the independent big toe representation seen in humans resulted from bipedal walking, which is suggested to have exerted strong selective pressure, for example, on foot morphology [5].

### (c) Hand and foot adaptation in the human ancestors

Existing modern and fossil evidence is insufficient to unambiguously establish the morphologies of the hands and feet at the various stages of human evolution. However, the accumulated hominin fossils do provide us with an informative evolutionary sequence of hand and foot structures. This can then be integrated with the above neurological results.

The human–chimpanzee bifurcation is most commonly estimated at 5–6 Ma [2,43,44], although an earlier divergence of greater than 7–9 Ma has been suggested [9,45] and is increasingly gaining support [46–48]. The deeper divergence hypothesis is in accord with the limited but persuasive hominin fossils known at 6–7 Ma [49–51]. Owing to lack of an informative fossil record surrounding the human–chimpanzee or (human + chimpanzee)–gorilla split, parsimony analysis and/or thinking lends support to a chimpanzee- or extant African-ape-like LCA [3–5,16]. However, deriving the human ancestral model from living chimpanzees is disputable [52]. Although living chimpanzees are our closest genomic neighbours their ancestors are as yet unidentified, so we cannot know how closely they resemble the LCA of humans and *Pan*. A cautionary note comes from the molecular evidence which reveals that humans are genetically more similar to the gorilla than to the chimpanzee in approximately 15% of the genome that differs between the three [44]. We argue below that the neuroscientific perspective of this study suggests a larger role for a quadrupedal model in elucidating human evolution.

Until recently, the only substantial body of information regarding the pre-*Homo* hominin hand and foot anatomy was provided by *Australopithecus* (approx. 4.2–1.4 Ma). An extensive review of this material is outside the scope of this discussion, but the most salient evidence pertaining to the results of this study is as follows.

The available hand element fossils of *Australopithecus* (*Au.*) *anamensis*, *Au. afarensis* and *Au. africanus*, from the 4.2 to 2.5 Ma time period, are seen by many to conform to a chimpanzee- or extant African-ape-like LCA model from parsimony [3–5]. Compared with a chimpanzee-like ancestral condition, these hominins had considerably shorter fingers and longer thumbs, suggesting better manipulative abilities. At the same time, *Australopithecus* hands are considered to share several features with the extant apes, such as a gracile thumb and various details of articular, carpal and phalangeal morphologies [3,4]. These commonalities are seen to support the hypothesis that *Australopithecus* retained a substantial degree of arboreality and climbing behaviour [3–5].

Hominin hand bones from the 2.0 to 1.5 Ma period have been reported to be more derived [4–6]. These fossils exhibit features such as a more robust thumb, flatter articulation between the thumb metacarpal and carpal bone and a suite of other morphologies considered to be associated with greater dexterity and gripping force. These advanced morphologies are often linked to enhanced stone-tool manufacture and use [4–6].

Post approximately 4.0 Ma, *Australopithecus* species are considered by most to have been adapted to bipedal locomotion, with a fully adducted non-opposable big toe and longitudinal arch [53,54]. However, many think they were still dependent on tree-climbing, citing morphologies of the hand and foot that differ from later *Homo* and are hence potentially more ape- or chimpanzee-like [55]. To these and other authors, the progressive morphological evolution of the hand, seen initially in *Australopithecus*, and especially after approximately 2.0 Ma, parallels progressive refinement of bipedality and the successive freeing of the hand from arboreal locomotor demand [4–6].

### (d) Insights from the recently discovered *Ardipithecus ramidus:* combining bipedality with quadrupedal climbing and aboreal clambering

The partial skeleton and other fossils of the approximately 4.4 Ma *Ar. ramidus* provide us with a somewhat different perspective. They include well-preserved hand and foot elements that enable evaluation of key functional structures [9–11], and are therefore the most important fossil evidence so far available in inferring the early evolutionary history and origins of the human limb. In the 2009 series of papers that reported this fossil material in some detail, it was suggested that *Ar. ramidus* represents a primitive adaptive evolutionary grade that preceded *Australopithecus* [9]. The known approximately 6 Ma fragmentary hominin fossils are also broadly comparable to *Ar. ramidus* in adaptively relevant morphologies [9], and probably also represent the *Ardipithecus* anatomy and behaviour. *Ardipithecus ramidus* therefore bridges the LCA to the more advanced *Australopithecus* and *Homo*, and enables new insights into both [9].

Compared with that of chimpanzees, the hand of *Ar. ramidus* had shorter fingers and a much shorter palm. Together with lack of carpal and metacarpal articular ‘strengthening’, the *Ar. ramidus* hand was not specialized for suspensory locomotion and it lacks the suite of knuckle-walking features seen in the African apes [10]. In short, in some key anatomical features, the *Ar. ramidus* hand was far from being extant chimpanzee-like, but better resembled both *Australopithecus* on the one hand, and the palmigrade Miocene apes and Old World monkeys on the other [10,12]. *Ardipithecus ramidus* indicates that the primitive hand morphologies shared by *Australopithecus* and apes [3,4] were actually not exclusively chimpanzee-like, but probably general primitive retentions.

The foot of *Ar. ramidus* has a fully opposable and grasping big toe for climbing, comparable with that of Old World monkeys and extinct and extant apes, whereas the lateral mid- to forefoot show evidence of some rigidity and phalangeal dorsiflexion for toe-off in bipedality [11]. As with the hand of *Ar. ramidus*, which was less specialized than that of the chimpanzee, a somewhat rigid lateral mid- to forefoot was probably a primitive retention from a Miocene ancestral condition. Together with the pelvic evidence [13], *Ar. ramidus* is interpreted to be a transitional biped that retained considerable arboreal capacities while being fully bipedal with extended hips and knees as in later hominins [9,11,13].

Seen from *Ar. ramidus*, the anatomical acquisition in *Australopithecus* of a non-opposable adducted big toe and longitudinal arch, which would have resulted in a remarkable forfeiting of arboreal capacities, must have been a highly significant evolutionary event [9]. Acquisition of a non-climbing foot probably signalled a major ecological transition in which terrestrial bipedal locomotion became more important than arboreal capacities. This is despite some apparent residual variation in *Australopithecus* foot fossils that suggest some degree of arboreal behaviour [55]. Such a scenario supposes a rapid transition of foot anatomy at approximately 4.2–4.4 Ma or earlier, depending on the timing of the cladogenetic event between *Australopithecus* and *Ardipithecus* [9]. This drastic structural change of the foot must have preceded changes in the *Ar. ramidus* to *Australopithecus* hand, which would have probably involved some finger shortening, thumb elongation, and hand broadening [10]. These changes most likely occurred sometime, or progressively, between approximately 4.4 and approximately 2.0 Ma, although not enough fossils are available for actual resolution in the timing of changes. Other morphological changes appear to have occurred with enhanced tool use after approximately 2.0 Ma [4,6].

### (e) Possible scenario of evolution of habitual bipedality, manual dexterity and tool-use

Penfield & Rasmussen [19], in his seminal brain-mapping studies, found that in 400 human patients, only 10 reported sensation confined to the toes during direct electrical stimulation on the cortex [19]. Sensation was reported in the big toe five times, in toe V once, and in all of the toes four times. Since then, the medial feet regions of SI have been virtually unexplored. Thus, prior to our study, the only representations that had been reported in humans were those for the big and fifth toes, whereas separate representations of the middle three toes had merely been inferred. We found that representations of the lesser toes were indistinct from one another. Monkeys exhibited fused SI representation for all five toes, unlike humans with a separate big toe. Although using different methods across species (humans by fMRI, and monkeys by single neuron recordings), this difference would be well justified as monkeys’ fused toes are revealed by the methods with higher spatial resolution whereas humans’ separate big toe is detected by fMRI with lower spatial resolution. Then, the results would suggest that acquisition of independent neurophysiological control of the big toe probably occurred sometime between the time of the LCA to that of *Australopithecus*. One possibility is that it occurred in *Ar. ramidus*, which did not toe-off with the big toe, but could have used it in balance control during stance phase. Another possibility is that it first occurred in *Australopithecus*, in parallel with acquisition of the adducted big toe and more human-like bipedality. In either case, it would have been associated with development of bipedal locomotion *per se*, and (largely or completely) independent of dexterity.

Our results also showed that advanced dexterity of the human hand is underlain by independent somatotopic representations of the fingers, but that this was shared with Old World monkeys, and hence probably a retention from an ancient arboreal quadrupedal ancestry. In the ancestral arboreal habitat, there was probably little need for the use of tools beyond, perhaps, simple use of branches [56]. Manual dexterity in quadrupedal higher primates may therefore have evolved primarily in response to the need to forage and ‘steer’ in a complex arboreal milieu. The hominin fossil record after approximately 4.0 Ma does progressively show anatomical evidence for enhanced manual function and thumb use, but these changes are less conspicuous compared with the structural changes seen in the foot. Therefore, it is possible that dexterous fingers were exapted at the preparatory stage in the ancestral anthropoid or catarrhines which resulted in later evolution of tool-use. The discovery of adaptive usages of stone tools to open and eat oysters by macaque monkeys in their natural island habitats at the Andaman Sea coast [57–60] demonstrates that such latent capabilities can flourish in certain environmental conditions, although it rarely happens. Axe hammering of those macaques even seemed to be a more precision type of tool handling than what chimpanzees are doing in their pound hammering [58].

What, then, would be a critical factor, in addition to presently shown hand dexterity, for hominins to exhibit spontaneous and universal usages of complex tools? A number of aspects of neural organization that influence brain–behaviour interactions which have not been examined in this study (such as, comparative cytoarchitectonic differences, differences in cortical connectivity, differences in the relative size of the cortical areas, etc.) might account for it. One such candidate may be the emergence of a human-specific tool-use cortical area. An area that could be this has recently been discovered using the same fMRI and visual stimuli for humans and monkeys [61]. It could support emerged neural mechanisms which boost, beyond the exapted fundamental ability, the sensory motor and cognitive functions required to understand the abstract causal relations involved in advanced tool-usages that are unique in humans.

## 5. Conclusion

The present findings suggest that ancestral primate hands and feet evolved (largely) independently. Contrary to received wisdom, one did not lead to the other. The comparative functional neurophysiological results presented above suggest an evolutionary role of brain–body interactions. The human foot realized upright bipedal walking through fundamental modifications of both shape and neurological function. The human hand, based on an ancestral functional principle and neurological substrate, achieved its fine dexterity by extending its shape and function much less drastically. The retention in humans of hand features from the ancestral arboreal monkey suggests living monkeys can be useful for studying tool-use mechanisms, which could have comprised a necessary condition for its later evolution. The evolutionary intensification of tool-use may include the integration of visual information [32] and symbolic/abstract information processing [61], leading to an emergence of a novel functional brain area for conceptual understandings of tool functions, fulfilling the sufficient condition for the boost of complex human tool-usages [62]. These mechanisms will be addressed in future studies.

## Notes

For human subjects the experiment was approved in advance by the Third Research Ethics Committee of RIKEN and was conducted in accordance with the Declaration of Helsinki. For the Monkeys the study was approved by the Animal Research Committee at the Toho University School of Medicine. All animal care and experimental procedures were in accordance with NIH guidelines for the care and use of laboratory animals.
